# Spindle Cell Lipoma of the Fourth Metatarsal: A Case Report

**DOI:** 10.7759/cureus.77220

**Published:** 2025-01-10

**Authors:** Khadijah Moustafa, Slava Albaghli, Selim Sürer

**Affiliations:** 1 Department of Pathology and Laboratory Medicine, King Saud Medical City, Riyadh, SAU; 2 Department of Medicine and Surgery, Ankara Yildirim Beyazit University, Ankara, TUR

**Keywords:** benign tumor, lipoma, metatarsal, neoplasm, spindle cell lipoma

## Abstract

Spindle cell lipoma (SCL) is a rare subtype of lipoma that typically arises in the subcutaneous tissue of the posterior neck and shoulders, most commonly in middle-aged men, and only rarely occurs in the extremities. SCL typically presents as a well-circumscribed, firm mass composed of CD34-positive adipocytes mixed with bland spindle cells and ropy collagen fibers. We present the case of a 46-year-old man who came to the hospital for routine hemodialysis treatment, during which his physician discovered a mass on his left fourth metatarsal that had been slowly growing for the past five years. A subdermal soft tissue lesion (5.6 cm x 3.8 cm x 4.5 cm) was excised, and histological findings confirmed the diagnosis of SCL. This case reports a rare occurrence of SCL in the metatarsal and emphasizes the importance of distinguishing SCL from its malignant equivalents, particularly when it occurs in rare and unusual locations.

## Introduction

Spindle cell lipoma (SCL), a subtype of lipoma, is a benign tumor characterized by spindle-shaped cells interspersed with mature adipocytes [1]. It accounts for 1-5% of all adipocytic tumors and is more common in men, with a male-to-female ratio of 10:1, typically occurring between the ages of 40 and 70. About 80% of SCLs are found in the subcutaneous tissue of the posterior neck, back, and shoulders, while the remaining 20% occur in other areas, such as the face or extremities [2,3]. We report a case of SCL in the fourth metatarsal, which was surgically amputated.

## Case presentation

A 46-year-old male visited the outpatient clinic for his regular hemodialysis session for end-stage renal disease (ESRD) when a painless mass on his fourth metatarsal was noted by his physician. The patient was unsure of how long the mass had been present but believed it to have been there and slowly growing over the past five years. There was no history of trauma, previous surgeries, or recurring lesions. No regional lymphadenopathy was identified. He also denied any symptoms of discharge, fever, or constitutional symptoms. The clinical diagnosis from the referring physician was lipoma or ganglion cyst. Examination revealed a sessile, well-circumscribed mass sitting at the plantar surface of his left fourth metatarsal. It appeared firm and non-fluctuant upon palpation. There was no pain, redness, or elevated temperature at the tumor site.

**Figure 1:**
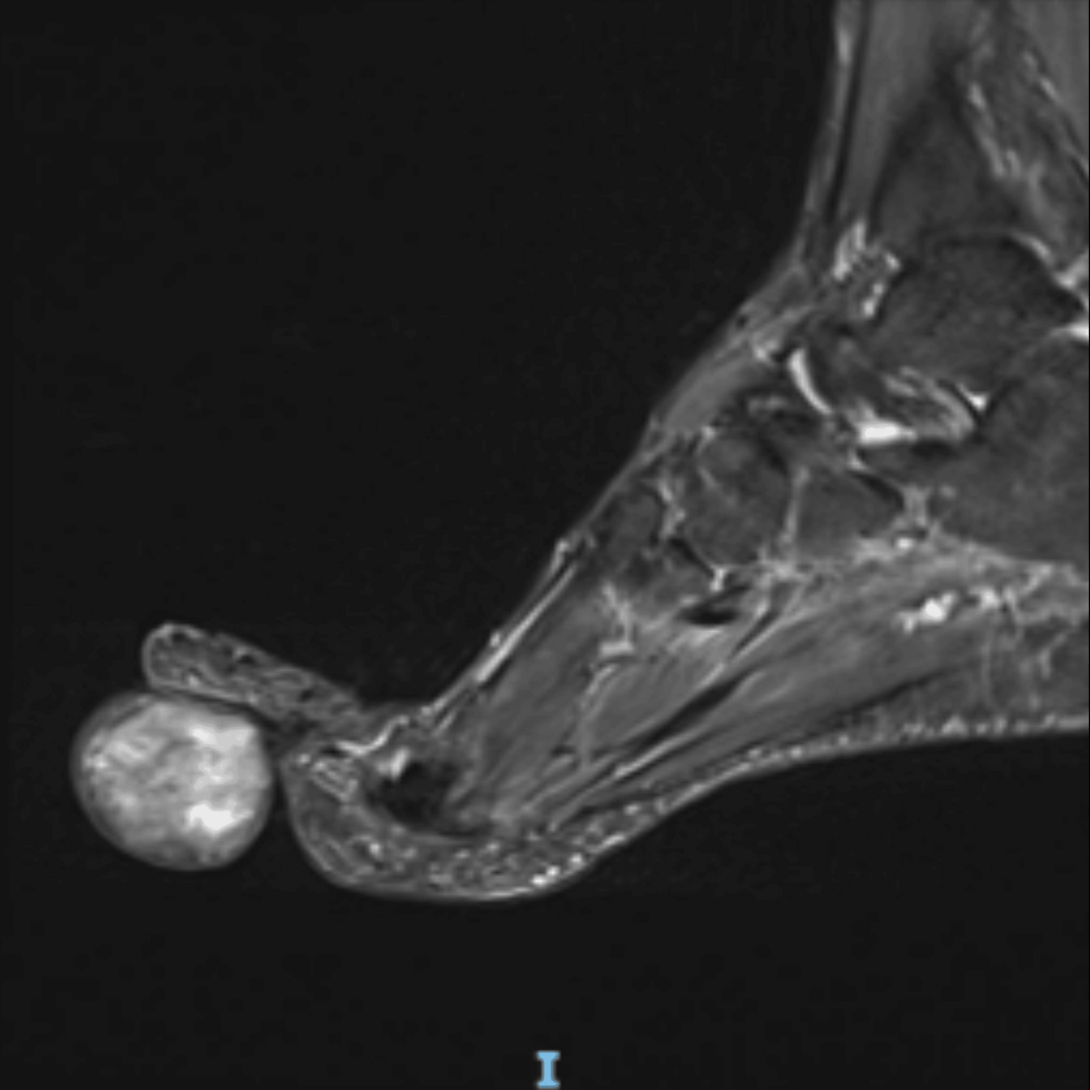
MRI imaging (sagittal section). T2-weighted images showing heterogenous hyperintensity signals.

A magnetic resonance imaging (MRI) scan was ordered and revealed a sizable oval/round subdermal soft tissue lesion (measuring 5.6 cm x 3.8 cm x 4.5 cm) that was partially abutting the distal fourth flexor digitorum tendon. It revealed an isointense signal on T1-weighted images with mild enhancement of its wall and hyperintense heterogenous signals on T2-weighted images (Figure 1).

**Figure 2:**
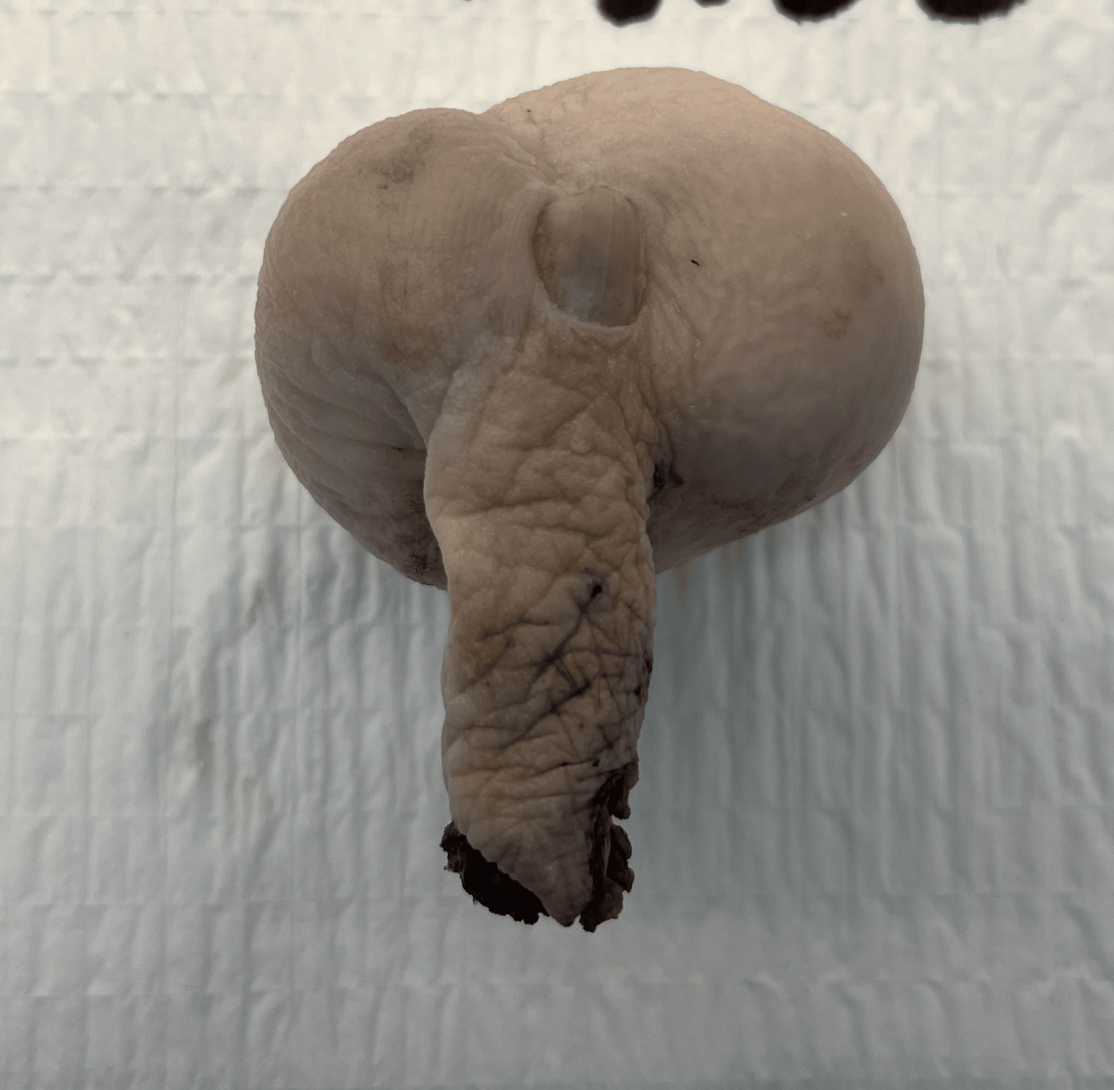
Anterior view of large mass in the fourth metatarsal.

**Figure 3:**
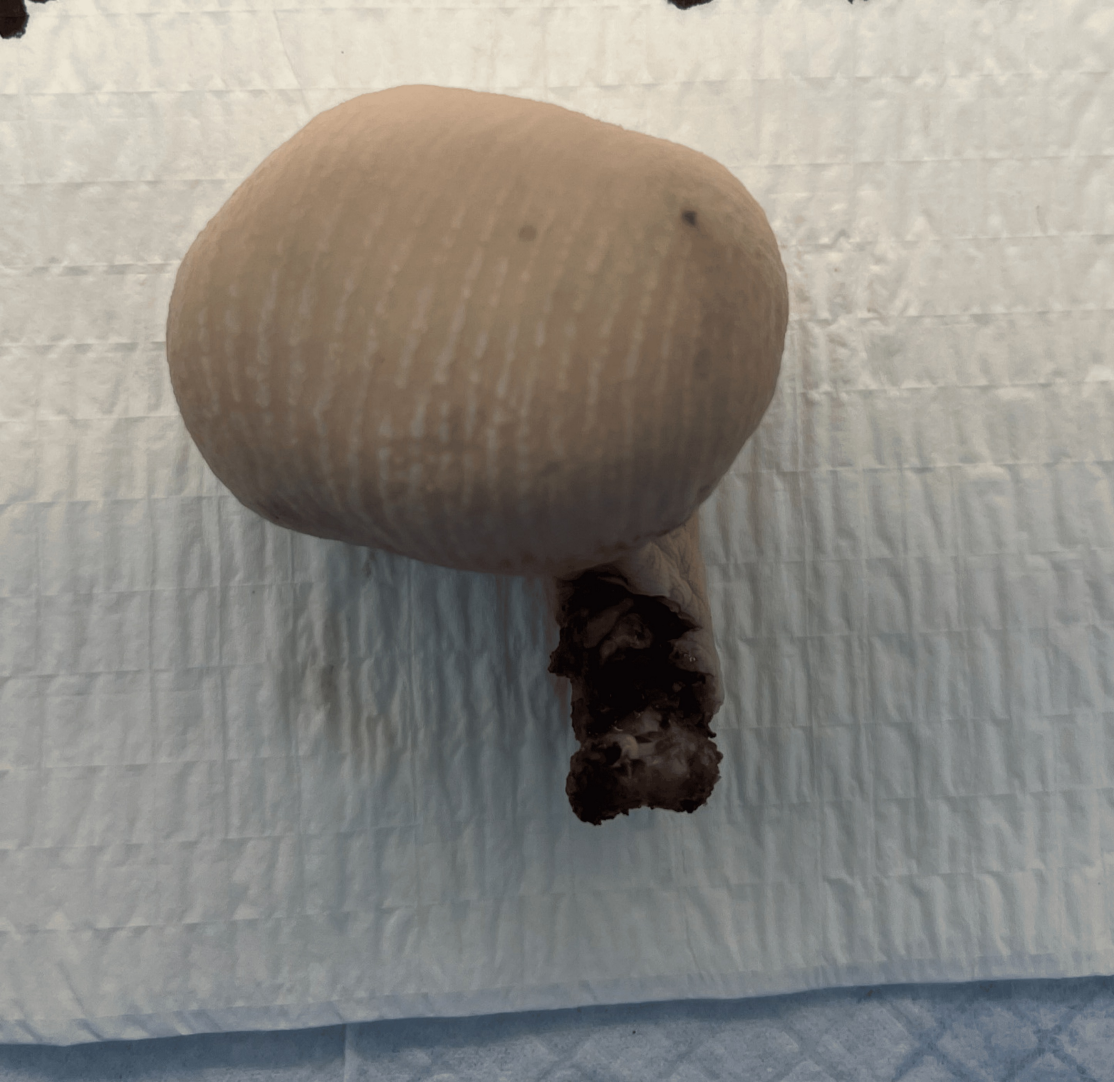
Posterior view of large mass in the fourth metatarsal.

The patient underwent Ray’s amputation of his fourth toe. The procedure was completed without any complications, and his post-op course was unremarkable. Grossly, the specimen consisted of a soft tissue mass and measured 5 cm x 4 cm x 3.5 cm, and the toe measured 4 cm x 2 cm x 0.5 cm (Figures 2, 3).

**Figure 4:**
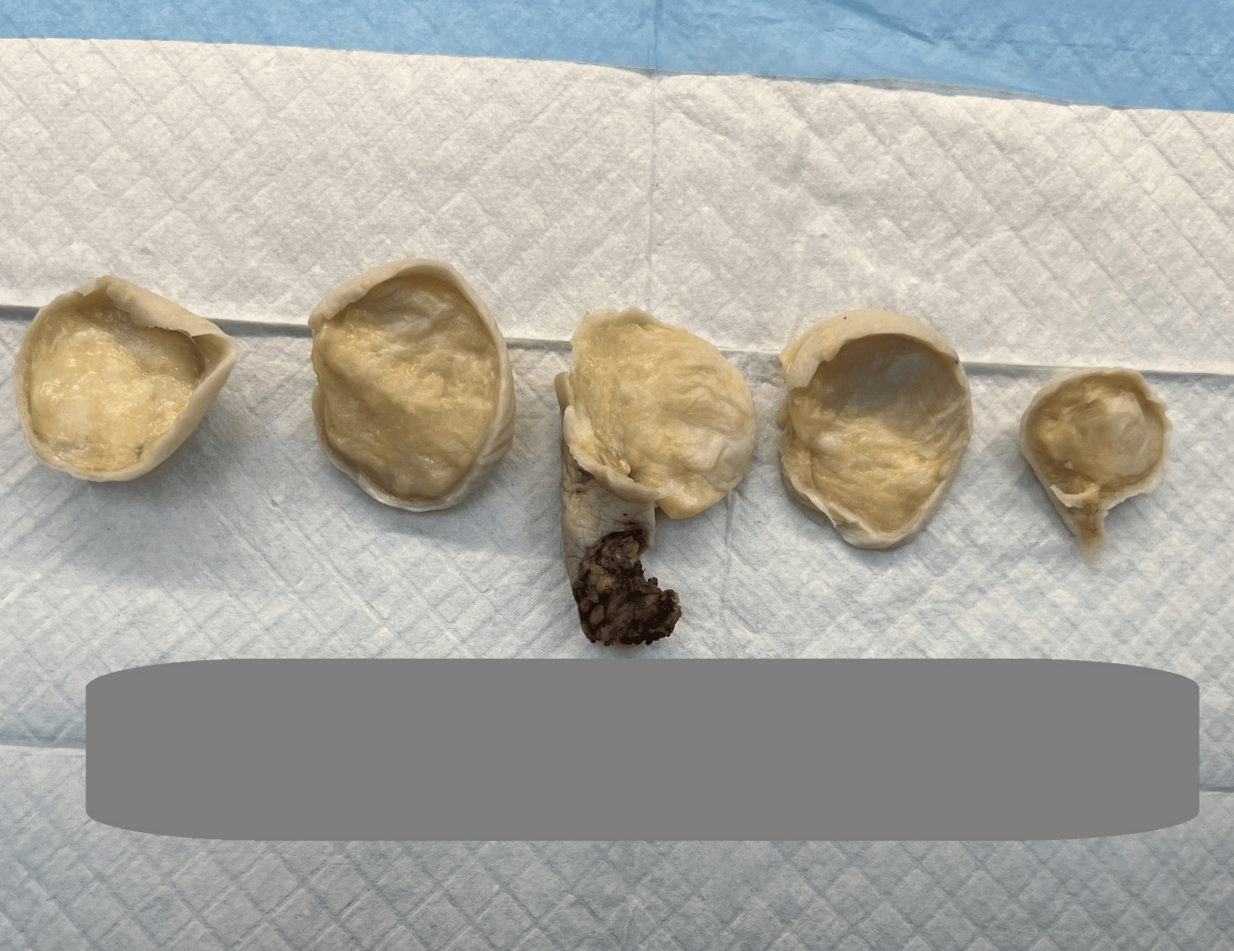
Cut section of mass revealing yellow, tan fatty cut surface.

The mass was 4 cm away from the bone resection margin. Serial sectioning of the mass revealed a yellow, tan fatty cut surface (Figure 4). There was no evidence of the lesion invading the bone.

**Figure 5:**
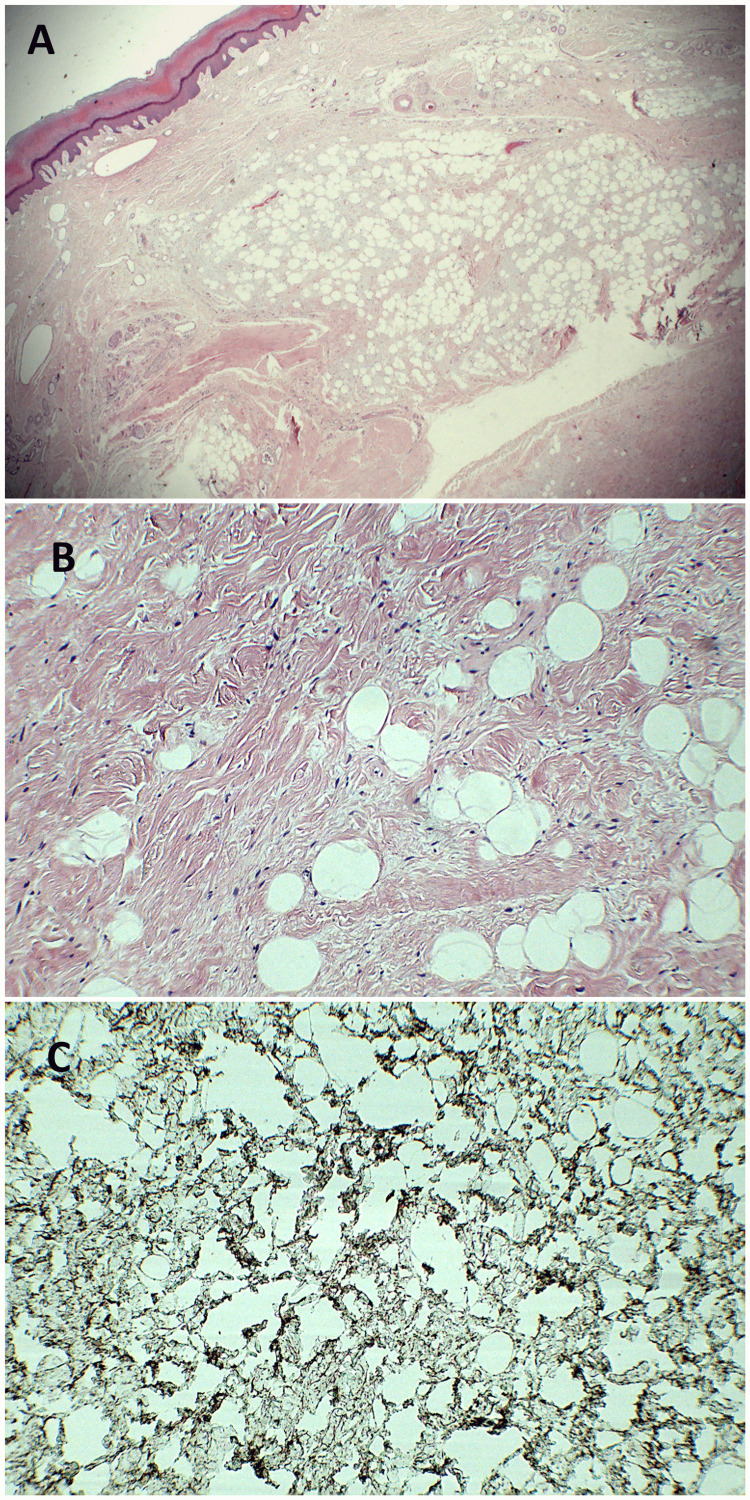
Pathological findings (hematoxylin–eosin stained after formalin fixation and paraffin embedding) shows proliferation of spindle cells with collagen fibers interspersed around mature adipocytic tissue. Original magnification: (A) X100 (B) X400 (C) positive staining for CD 34.

Histopathological examination of the specimen revealed an adipocytic neoplasm with spindle-shaped cells or pleomorphic lipoma. Immunohistochemical staining revealed that spindle cells are diffusely positive for CD34, further supporting the diagnosis of spindle cell lipoma (Figures 5A, 5B, 5C).

## Discussion

Lipomas are common benign soft tissue tumors composed of adipose (fat) cells surrounded by a thin layer of fibrous tissue. They occur in both adults and children, most frequently in the subcutaneous tissues of the head, neck, shoulders, and back. Among the various types of lipomas, spindle cell lipoma (SCL) is a rare subtype, accounting for only 1-5% of all lipomas [2]. Only 2% of SCLs are in the toe [3,4]. To the best of our knowledge, only four other cases of metatarsal SCL have been reported in the literature to date. These tumors are generally asymptomatic and do not require treatment unless causing discomfort due to their location or size. SCL typically occurs in men aged 40 to 70, predominantly in the posterior neck, back, and shoulders [2,4]. 

The pathophysiology of SCL is not fully understood. However, spindle cells are hypothesized to originate from various precursors, including fibroblasts, adipocytes, immature mesenchymal cells, and CD34-positive dendritic interstitial cells [1]. Immunohistochemical analysis of SCL typically shows spindle cells that are positive for CD34 and negative for S-100, which helps differentiate SCL from other lipomatous tumors, such as well-differentiated liposarcoma (WDL) [1,2]. Additional features like desmin negativity and chromosomal deletions on 12q and/or 16q can also aid in diagnosis [2,4].

The differential diagnosis includes several benign lipomatous tumors, as well as WDL, which often presents in areas similar to SCL and may have indistinct margins. SCL is distinguished by the uniform appearance of its spindle cells and the absence of lipoblasts, which are present in liposarcoma. Recurrent growth following excision may suggest a diagnosis other than SCL, as complete excision is typically curative [2,4].

Imaging, such as MRI, can be useful in assessing the extent and characteristics of SCL, although its features may overlap with those of other soft tissue tumors. With regard to SCL, 52% of patients have >50% but <90% fatty areas, while 19% have fatty areas below 50% [5]. In our case, MRI revealed a well-circumscribed mass involving the fourth metatarsal, suggestive of a benign soft tissue tumor. However, a definitive diagnosis required histopathological examination, which revealed a mixture of mature adipocytes and bland spindle cells in a characteristic collagenous pattern.

Treatment of SCL involves surgical excision, which is usually curative due to the tumor's benign nature. Wide resection margins are unnecessary, and recurrence is rare, although long-term follow-up is recommended to monitor for potential recurrence or malignant transformation [3,6]. Our case contributes to the limited literature on metatarsal SCL, showing the importance of recognizing this rare entity and confirming the diagnosis with appropriate histopathological and immunohistochemical studies.

## Conclusions

In conclusion, this case report highlights a rare presentation of spindle cell lipoma (SCL) in the fourth metatarsal, emphasizing the need for careful differential diagnosis, especially with unusual soft tissue masses in atypical locations. Spindle cell lipoma can mimic other malignant entities both clinically and radiologically. Histopathological examination, supported by immunohistochemical staining, remains the gold standard for diagnosis. Surgical excision with clear margins is the treatment of choice, and long-term follow-up is necessary to monitor for recurrence or malignant transformation. 
